# Hatha Yoga Practices: Energy Expenditure, Respiratory Changes and Intensity of Exercise

**DOI:** 10.1093/ecam/neq046

**Published:** 2011-06-15

**Authors:** Uday Sankar Ray, Anjana Pathak, Omveer Singh Tomer

**Affiliations:** ^1^Environmental Physiology Division, Defence Institute of Physiology and Allied Sciences, Defence Research and Development Organization, Timarpur, Lucknow Road, Delhi 110054, India; ^2^Work Physiology and Yoga, Defence Institute of Physiology and Allied Sciences, Defence Research and Development Organization, Delhi, India

## Abstract

The aim of this study was to critically observe the energy expenditure, exercise intensity and respiratory changes during a full yoga practice session. Oxygen consumption ($\dot{\text{V}}{\text{O}}_{2}$), carbon dioxide output ($\dot{\text{V}}{\text{CO}}_{2}$), pulmonary ventilation ($\dot{\text{V}}$E), respiratory rate (Fr) and tidal volume (VT), were measured in 16 physical posture (asanas), five yoga breathing maneuvers (BM) and two types of meditation. Twenty male (age 27.3 ± 3.5 years, height 166.6 ± 5.4 cm and body weight 58.8 ± 9.6 kg) yoga instructors were studied. Their maximal oxygen consumption ($\dot{\text{V}}{\text{O}}_{2\max}$) was recorded. The exercise intensity in asanas was expressed in percentage $\dot{\text{V}}{\text{O}}_{2\max}$ . In asanas, exercise intensity varied from 9.9 to 26.5% of $\dot{\text{V}}{\text{O}}_{2\max}$ . Highest energy cost was 3.02 kcal min^−1^. In BM highest $\dot{\text{V}}$E was 53.7 ± 15.5 l min^−1^. VT was 0.97 ± 0.59, 1.41 ± 1.27 and 1.28 ± l/breath with corresponding Fr of 14.0 ± 5.3, 10.0 ± 6.35, 10.0 ± 5.8 breaths/min. Average energy expenditure in asanas, BM and meditation were 2.29, 1.91 and 1.37 kcal min^−1^, respectively. Metabolic rate was generally in the range of 1-2 metabolic equivalents (MET) except in three asanas where it was >2 MET. $\dot{\text{V}}{\text{O}}_{2}$ was 0.27 ± 0.05 and 0.24 ± 0.04 l min^−1^ in meditation and Shavasana, respectively. Although yogic practices are low intensity exercises within lactate threshold, physical performance improvement is possible owing to both better economy of breathing by BM and also by improvement in cardiovascular reserve. Other factors such as psycho-physiological and better relaxation may contribute to it.

## 1. Introduction

There is literature on the ancient Indian system of yoga regarding its positive effects on various physiological systems. Raub [1] in a review has shown that psycho-physiological changes by yoga help in the improvement of both musculoskeletal and cardiopulmonary functions. Long-term yoga practice improves depth of breathing and alters chemoreceptive sensitivity [2, 3]. It also reduces metabolic rate in healthy subjects [4]. Meditation leads to hypo-metabolic state [5, 6], but specific respiratory exercises (pranayama) do the opposite [6]. Yoga improves physical performance [7, 8], body flexibility [9] and mental health [10]. Its therapeutic potentials in various diseases particularly for life-style-related ones have been explored and are being utilized. The four leading risk factors like overweight, high blood pressure, high blood glucose and cholesterol, which are linked to life-style-related chronic diseases, may be reduced by yoga intervention [11–14]. It helps in the reversal of coronary artery diseases [15]. It has even helped to improve physical performance and to reduce the level of inflammatory markers in chronic heart failure patients with 25% ejection fraction [16]. Yogic practices are useful in the management of diabetes [17, 18] and bronchial asthma [19]. It has also been shown that if the intensity and duration of yoga training is not proper the expected effect of yoga may not be observed as in case of diabetic patients [20]. Yoga has its great psychological benefits by reduction of stress, anxiety and depression [10, 21–23]. Its role in the treatment of chronic insomnia has also been reported [24]. Yoga is rapidly gaining popularity. The number of people practicing yoga for health benefits in India as well as abroad has increased significantly in the past decade [25]. In spite of continued interest of the scientific community, there is still paucity of data on basic physiological responses related to yoga practices. The data on energy expenditure during its practice as well as relevant data to express the intensity of exercise in terms of exercise physiology or nutritionist have not been documented systematically as it has been done for various sports or other day-to-day activities or for occupational activities. “The compendium of physical activities” [26] a coding scheme that classifies physical activity based on energy expenditure does not include energy expenditure while performing various types of yoga. The literature in this regard either reported it by citing single yoga practice or by giving it in a more general way [27–38]. Kyeongra Yang [11] stressed this point in his review article. The report in this regard for a full yoga session has been provided only by four groups of workers [34–37]. First group is ours [34] that focused on Suryanamaskar (Sun salutation). Second group studied on women and on the mixed samples of a wider age range, that is, 19–40 years old [35]. Two studies [35, 36] reported on the yoga sessions which comprised of Surya namaskar as a major part of the entire session. Another was based only on six yoga positions [37]. These studies have given intensity of exercise in terms of percentage of maximal heart rate (%MHR) and on energy expenditure. However, none has evaluated the intensity of exercise in yoga postures in terms of each subject's percentage of actual maximal oxygen uptake capacity (% $\overset{.}{\text{V}}{\text{O}}_{2\max\;}$). Hence, the true relative intensity of physical activity could not be revealed. In view of the evidences that in yoga %MHR overestimates the corresponding level of oxygen consumption [37], a study incorporating % $\overset{.}{\text{V}}{\text{O}}_{2\max\;}$ has been long due. Moreover, no study is available where the parameters like ventilatory equivalent for oxygen and carbon dioxide, pulmonary ventilation have been provided with the oxygen consumption to evaluate the yogic exercises in terms of exercise physiology. The data in this regard will be helpful for nutritionist, therapists as well as researchers involved in this field. It will also be helpful for the people involved in various yoga programs to get an idea of energy expenditure and intensity of exercise of different yoga practices for incorporating them in yoga sessions according to the need. In this background, the aim of this study was to evaluate the energy expenditure during a full session of yoga practice on professional male yoga practitioners of comparatively shorter age range and to express its intensity in terms of metabolic equivalent (MET) and % $\overset{.}{\text{V}}{\text{O}}_{2\max\;}$ for each of its components individually. The study also noted the behavior of other relevant respiratory parameters.

## 2. Methods

### 2.1. Subjects

Twenty healthy male yoga practitioners/instructors who had been practicing yoga for 6–10 years volunteered for the study. Their mean age, height, body weight, body fat percentage and lean body weight (LBW) were 27.4 ± 3.45 years, 166.6 ± 5.4 cm, 58.8 ± 9.62 kg, 10.9 ± 3.1 and 52.2 ± 2.8 kg respectively.

All subjects of this study were of one gender that is, only male instead of both male and female. This is due to the scientifically well-accepted fact that the male and female have different range of physiological parameters in normal condition. Again, the magnitude of the effect of physical training on them also differs. This happens due to different hormonal profile of two genders. There was a study [4] that showed that magnitude of response to basal metabolic rate by yoga training in male and female volunteers was different. In one of our previous study [10] also, in view of gender differences, the data of male and female subjects were presented separately. Therefore, to get a better resolution of the facts only one gender (male) was selected. Again, subjects selected for this study were experienced yoga practitioners/instructors. This was due to the following facts. The main aim of this study was to have some standard values with respect to different asanas and other yogic practices. Earlier in a same type of study (unpublished) recordings were taken by us during yoga training in two phases on people who were novice in yoga practice. The limitation of that study was the variations in physiological responses based on the subjects' daily activity pattern; if they were athletic type their energy expenditure during yoga practice was lower than sedentary individuals. So, again it may vary to a certain extent in different populations based on the respective physical activity level. As there were no data available in this field where similar type of study had been done, preference was given more to provide the data with respect to those people (yoga instructors) who were supposed to be more accurate and perfect in performing yoga postures/practices. These subjects were supposed to have more stable responses. Thus, it also helped to exclude the effects those likely to have in semi-trained or untrained persons.

### 2.2. General Protocol

In this study only one group of subject who practiced only yoga was considered (i.e., “Yoga" group). Another group, that is, “control" who would have practiced other form of conventional exercises was not included. The reason behind this was the very purpose of this study where measurements of various physiological parameters needed to be done during actual yoga practice. However, logically the people of the “control group" who would have been trained in aerobics in a conventional way could not have been trained in yoga. So, they could not have practiced yoga. Thus, it would not have been possible to get their values during the actual yoga practice. If they also would have practiced yoga, they could not have been treated as “control" group (it would have been a mixed yoga and non-yoga-aerobics group).

Subjects were explained about the various tests, the purpose of the study and the risk involved as in maximal exercise test. They were also made aware of the precautions taken thereof. They gave their informed consent to participate in the study and the ethical committee of the institute who scrutinizes the standards with respect to the ethical principles for research involving human subjects approved the test protocol. Test trial of the protocol was given to each individual few days prior to the day of actual test to acquaint them with the procedure so as to avoid anxiety that would have otherwise interfered with the results. It also helped in the standardization of the experiment.

### 2.3. Yoga Practices

The study wanted to encompass greater variety of yoga postures/practices in its purview to get closer view of the behavior/range of various physiological parameters within a reasonable time frame to arrive at a realistic conclusion. Hence, a representative yoga practice schedule as usually been practiced by normal healthy person of this age group was considered.

**Figure 1:**
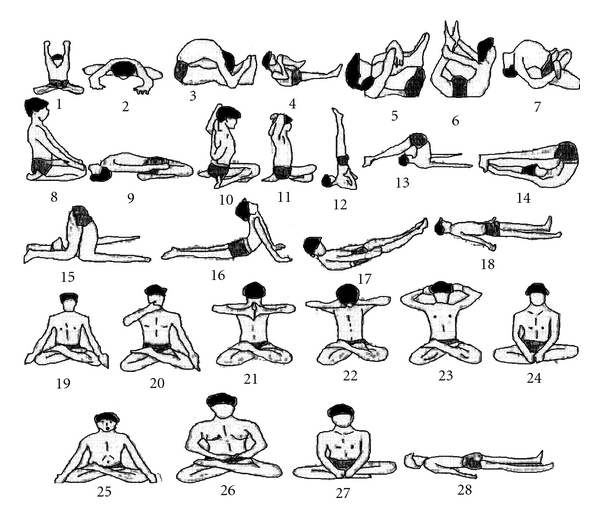
The various yoga postures as practiced by the subjects during a full yoga session. (1) Yoga mudra asana 1-YM 1, (2) Yoga mudra asana 2-YM 2, (3) Paschimottanasana-PSN, (4) Supta pavan muktasana-SPVM, (5) Pavan muktasana-PVM, (6) Dhanurasana-DHN, (7) Matsyasana-MYS, (8) Vajrasana-VAJ, (9) Supta Vajrasana-SVAJ, (10) Gomukhasana 1-GMK1, (11) Gomukhasana 2-GMK 2, (12) Sarvangasana-SARV, (13) Halasana 1-HAL1, (14) Halasana 2-HAL 2, (15) Karnapeedasana-KPED, (16) Bhujangasana-BHUJ, (17) Utthanpadasana-UTP, (18) Shavasana-SAV, (19) Kapalbhati-KB, (20) Bhastrika-BHAS, (21) Kaki mudra-KAKI, (22) Yoni mudra-YONI, (23) Bhramari pranayama-BHRA, (24) Sukhasana-SUKH, (25) Omkar meditation-OMMED, (26) Meditation-MED, (27) Sukhasana-SUKH, (28) Shavasana-SAV.

The subjects performed various yoga practices as listed in Table 1 and Figure 1 for about 1 h in the laboratory. The abbreviations with respect to different yoga practices are given in Figure 1. Various Yoga practice schedule consisted of hatha yoga asanas (various static physical postures), pranayama (breathing maneuvers—BMs) and meditation. Hatha yoga exercises were administered according to the standard procedure [39, 40]. Duration (in minutes) for each yogic practice as performed during the yoga session is also given in Table 1. The entire Yogic exercise schedule consisted of hatha yoga asana (24 min), BMs (8 min) and meditation (5 min). Usually after every asana adequate rest in the form of Shavasana (SAV) was given. During this time oxygen consumption ($\overset{.}{\text{V}}$O*˙*_2_) along with heart rate were monitored. Stability of these parameters by arriving at basal resting values was considered as indicator of adequate rest and washout in between asanas. This was done to get stable state to avoid the carry-over effect on the subsequent asanas. The basic requirement of any yoga program is also to provide adequate rest in the form of SAV in between asanas or other practices. 

### 2.4. Measurements

In general, measurements were conducted in two sets on two different days. First set was done during a full yoga session in the laboratory when measurements were conducted while subjects were performing the actual yogic practices. Second set was carried out on some other day when their maximal exercise test was conducted on an ergometer to get their maximal oxygen uptake capacity ($\overset{.}{\text{V}}{\text{O}}_{2\max\;}$) values. This was used to calculate intensity of exercise in terms of %$\overset{.}{\text{V}}{\text{O}}_{2\max\;}$ for different yoga postures.

#### 2.4.1. Anthropometry

Body weight of the subjects was recorded by a precalibrated human weighing machine (ATCO Electronic Platform Scale EW Series, India) and skin fold thickness was taken by Harpendon skin fold caliper (Holtain Ltd, UK). Body fat was calculated by the method of Sloan [41].

### 2.5. Rest and during Yoga Practice

Their $\overset{.}{\text{V}}$O*˙*_2_, ventilation ($\overset{.}{\text{V}}$ *˙*E), respiratory rate (Fr) and tidal volume (VT) were recorded by oxygen-consumption measurement system (Model Oxycon Champion, Erich Jaeger, Germany), while they were actually performing the yoga practices after 30 min rest in supine position. The system was calibrated regularly for volume of air by a standard 2 l syringe and for oxygen and carbon dioxide percentage by standard gas mixture. During trial runs, days before the actual tests, approximate duration in between two asanas in SAV posture was determined by observing $\overset{.}{\text{V}}$O*˙*_2_ and heart rate to get stable state. In this way, approximate time period required for stabilization by achieving basal values of those parameters (recovery) was determined after each yoga practice for better guidance during the day of actual experiment. Based on this and also by studying in the same way during the actual day of experiment the duration between various practices were taken into consideration. For Bhastrika (BHAS), the breathing mask was removed for the time being during its practice as with the mask it was not possible to perform these maneuvers properly. Recording was taken just after its practice. All the tests were conducted in the forenoon (09:00–11:00 h). Temperature of the laboratory was maintained at 26–28°C and humidity at 60–80% during the period of experiment.

### 2.6. Maximal Exercise Test

Subjects performed maximal exercise on a bicycle ergometer (Ergoline, Gmbh, and ergometrics ER 900, Germany) on a day other than the day when they practiced yoga. Their $\overset{.}{\text{V}}$O*˙*_2_, $\overset{.}{\text{V}}$E*˙*, Fr and VT were measured by oxygen consumption measurement system (Oxycon Champion, Erich Jaeger, Germany). Heart rate was monitored from ECG recording (MX lead by the same system). They pedaled at zero load for 5 min as warm up exercise. Then, they were given a brief rest while sitting on the ergometer. Subsequently, when heart rate and $\overset{.}{\text{V}}$O*˙*_2_ were in steady resting level, they were administered exercise in a graded exercise protocol starting from 25 W workload and progressively increasing it by 25 W every minute (at 60 rpm) until exhaustion. Exercise was stopped when subjects could not keep the rhythm of pedaling and $\overset{.}{\text{V}}$O*˙*_2_ reached a plateau.

### 2.7. Data Analysis

For analysis and comparison, different yoga practices were grouped as separate clusters. First cluster was having various asanas (static physical postures). Second cluster was having various BMs, that is, Kapal Bhati (KB), BHAS, Kaki Mudra (KAKI), Yoni Mudra (YONI) and Bhramari pranayama (BHRA). The third cluster consisted of various meditative practices along with the postures in which meditation is practiced when $\overset{.}{\text{V}}$O*˙*_2_ was supposed to be in the lower range, that is, Omkar meditation (OM MED), Meditation (MED), SAV and Sukhasanas (SUKH). For the calculation of MET, the ratio of $\overset{.}{\text{V}}$O*˙*_2_ required during yoga practice to the $\overset{.}{\text{V}}$O*˙*_2_ at rest was considered. The energy cost of each yoga practice was derived using $\overset{.}{\text{V}}$O*˙*_2_ corrected to non-protein respiratory quotient [42]. The amount of oxygen required for a person for a particular activity in terms of percentage of his maximum ability to consume oxygen ($\overset{.}{\text{V}}{\text{O}}_{2\max\;}$) is expressed as % $\overset{.}{\text{V}}{\text{O}}_{2\max\;}$. Only first cluster (asanas) have been considered to express intensity of exercise in terms of $\overset{.}{\text{V}}{\text{O}}_{2\max\;}$ as in the rest of the yoga practice $\overset{.}{\text{V}}$O*˙*_2_ was very low.

### 2.8. Statistics

Statistical analysis of the data was performed by two-way classification of analysis of variance technique using the Newman-Keuls multiple range test to compare the same group at different situations.

## 3. Results

### 3.1. $\overset{.}{\text{V}}$O*˙*_2_

Changes in $\overset{.}{\text{V}}$O*˙*_2_ and other parameters are given in Tables 2, 3 and 4. The $\overset{.}{\text{V}}$O_2_ value during the practice of Pavan muktasana (PVM), Dhanurasana (DHN), Sarvangasana (SARV), Halasana 1 (HAL1), Halasana 2 (HAL2) and Karnapedasana (KPED) were 0.542 ± 0.12, 0.624 ± 0.07, 0.562 ± 0.14, 0.571 ± 0.18, 0.631 ± 0.19 and 0.571 ± 0.17 l min^−1^, respectively, and those were of significantly higher range as compared with other asanas. In Matsyasana (MYS), Gomukhasana (GMK2), Bhujangasana (BHUJ) and Uttangpadasana (UTP) it was 0.471 ± 0.12, 0.521 ± 0.10, 0.471 ± 0.08 and 0.502 ± 0.10 l min^−1^ respectively. It was significantly higher (*P* <  .01) in PVM as compared with Supta Pavan Muktasana (SPVM). Value of Supta Vajrasana (SVAJ) was higher (*P* <  .05) than Vajrasana (VAJ). In SAV it was 0.243 ± 0.04 l min^−1^. Other than SAV lowest value was observed in Yoga Mudra (YM2), that is, 0.369 ± 0.07 l min^−1^. In PSM, SPVM, VAJ, SVAJ and GMK1 it varied from 0.372 to 0.433 l min^−1^. Among the yogic BMs highest values were 0.514 ± 0.16 and 0.503 ± 0.18 l min^−1^ in KAKI and YONI, respectively. Value of KB was 0.451 ± 0.15 l min^−1^. BHAS showed lowest value, that is, 0.262 ± 0.04 l min^−1^. Other than BHAS and BHRA, values of all other yogic BMs were significantly (*P* <  .01) higher than SUKH. Value of OM MED, that is, 0.301 ± 0.08 l min^−1^ was significantly (*P* <  .01) higher than SAV, that is, 0.243 ± 0.04 l min^−1^. Value of MED was 0.272 ± 0.05 l min^−1^ and it was not significantly different from OM MED.

### 3.2. Energy Cost

DHN and HAL2 showed significantly higher energy cost among asanas, that is, 3.03 and 3.05 kcal, respectively. In KB, KAKI and YONI values were significantly higher among BMs and in BHAS and BHRA it was lower. Energy cost in SUKH (1.49 kcal) significantly reduced in MED (1.29 kcal). Energy cost also reduced from OM MED to MED to SAV. Total energy expenditure of this program was 55.45 kcal (asanas 41.2 kcal, BMs 11.50 kcal and meditation 2.75 kcal).

### 3.3. Intensity of Exercise

#### 3.3.1. %$\overset{.}{\text{V}}{\text{O}}_{2\max\;}$

**Figure 2:**
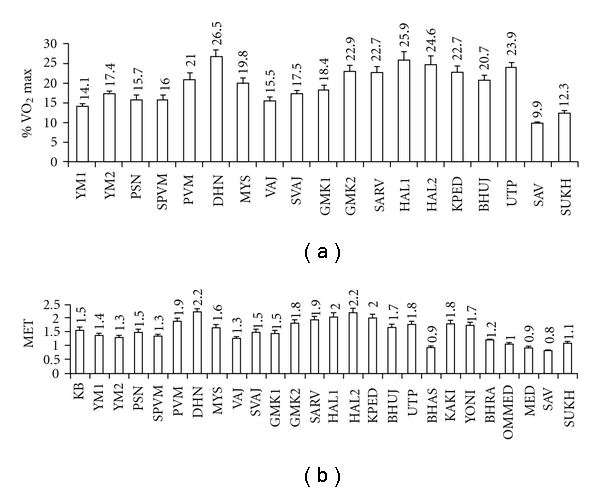
Intensity of exercise in terms of percent aerobic capacity ($\overset{.}{\text{V}}{\text{O}}_{2\max\;}$) in different asanas and MET in full yoga session (values are mean ± SEM).

As given in Figure 2 intensity of exercise in terms of %$\overset{.}{\text{V}}{\text{O}}_{2\max\;}$ among asanas was in highest range in DHN (26.5%), HAL-1 (25.9%), and HAL-2 (24.6%) and in UTP (23.9%). It was in the lower range in YM1 (14.1%), YM2 (17.4%), Paschimottanasana (PSN) (15.7%), SPVM (16.0%) and in VAJ (15.5%).

#### 3.3.2. MET

Most of the yoga practices were in the range of 1-2 MET. Exception was in DHN, HAL-1 and HAL2 where values were of >2 MET. In KB, KAKI and YONI values were in higher range among BMs. Lowest value was in BHAS. In SUKH it was 1.08 which significantly reduced to 0.93 (*P* <  .05) in MED. Sequentially, it was lower from OM MED to MED to SAV (*P* <  .01). There was no significant difference between OM MED and SUKH and also between MED and SAV but it significantly (*P* <  .05) reduced from SUKH to MED.

#### 3.3.3. $\overset{.}{\text{V}}$E

Among the asanas, highest $\overset{.}{\text{V}}$ *˙*E was observed in DHN (17.9 ± 2.32 l min^−1^). It was significantly higher than other asanas except HAL-1 (16.9 ± 3.79 l min^−1^), HAL2 (16.7 ± 3.24 l min^−1^) and UTP (16.1 ± 3.34 l min^−1^). In SAV and YM2, mean value of $\overset{.}{\text{V}}$ *˙*E was lowest, that is, 6.9 ± 1.43 and 9.3 ± 1.90 l min^−1^ respectively and those were significantly (*P* <  .01) lower among asanas except VAJ. In other asanas, values were in the range from 9 to 15 l min^−1^. Among the yoga BMs $\overset{.}{\text{V}}$E was significantly (*P* <  .05 to *P* <  .01) higher in KB (53.5 ± 15.47 l min^−1^) than BHAS, KAKI, YONI, BHRA (values were in the range of 6.6–12.7 l min^−1^). Mean value of OM MED (8.5 ± 2.4 l min^−1^) was significantly (*P* <  .05) higher than MED (6.9 ± 1.62 l min^−1^).

#### 3.3.4. Fr

Values in HAL1, HAL2 and KPED were 27.0 ± 4.2, 27.0 ± 6.2 and 27.0 ± 6.3 breaths/min respectively and were significantly higher (*P* <  .01) than all other asanas except SARV and PVM. Fr in SARV (25.0 ± 5.2 breaths/min) was also significantly higher (*P* <  .01) than that of other asanas except PVM, DHN, UTP and GMK2. In PSN, PVM, MYS, SVAJ, GMK1, GMK2 and BHUJ it was in the range from 17 to 24 breaths/min. It was significantly higher (*P* <  .05) in PVM as compared with SPVM. Among BMs highest and lowest values were observed in KB (65 ± 27.4 breaths/min) and in BHRA (10.0 ± 6.35 breaths/min), respectively. Values of Fr in KB were significantly higher (*P* <  .01) than other BMs. Lowest value was observed in OM MED (10.0 ± 5.82 breaths/min) among MED, OM MED, SAV and SUKH and it was significantly (*P* <  .01) lower than SUKH.

#### 3.3.5. VT

Among asanas, lowest VT was in KPED, that is, 0.57 ± 0.11 l/breath and it was significantly lower than YM1 (*P* <  .01), YM2 (*P* <  .01), MYS (*P* <  .05). Values of VT in PSN, SARV, HAL1, HAL2 and DHN were in the range of 0.61–0.73 l/breath. In BHRA (1.41 ± 1.27 l/breath) it was significantly (*P* <  .05 to *P* <  .01) higher than other BMs. Value of OM MED (1.28 ± 1.14 l/breath) was significantly (*P* <  .01) higher than MED, SAV and SUKH.

### 3.4. Ventilatory Equivalent for Oxygen

Among asanas highest value was observed in UTP (27.8 ± 5.3), which was significantly higher than all other asanas. Lowest value was observed in YM2, that is, 21.2 ± 3.53. In rest of the asanas, ventilatory equivalent for oxygen (EQO2) was in the range of 22.8–26.8. Highest EQO2 was observed in KB, that is, 79.0 ± 33.6 (*P* <  .01) compared with other yogic BMs and SUKH. EQO2 in OM MED was 25.2 ± 6.4, which was significantly higher (*P* <  .05) than MED.

### 3.5. Ventilatory Equivalent for Carbon Dioxide

The highest value was observed in GMK1 (30.5 ± 3.8). Values of YM1, YM2, SARV, HAL2 and KPED were in the same range. Values of SVAJ (29.8 ± 3.6), GMK2 (29.9 ± 3.7), VAJ (29.8 ± 3.5), SPVM (29.1 ± 4.3), PVM (29.0 ± 3.95), DHN (28.4 ± 5.8), MYS (28.9 ± 3.8), were next to the highest range. Lowest value was in YM2 (26.5 ± 2.54) and it was significantly lower (*P* <  .01) than other asanas except YM1, PSN, SARV, HAL2 and KPED. Ventilatory equivalent for carbon dioxide (EQCO2) in KB was significantly higher (*P* <  .01) than that of BHAS, KAKI, YONI, BHRA and SUKH. 

## 4. Discussion

Intensity of exercise of the yoga practices in this study was in the range from 9.9% (SAV) to 26.5% (DHN) of $\overset{.}{\text{V}}{\text{O}}_{2\max\;}$ (Figure 2). In general, it was in the lower range of sub-maximal level of exercise in most of the asanas except in SAV where it could be considered as in the resting level. It is also directly related to the greater involvement of active muscle mass from the different parts of the body as in PVM. In PVM both legs are involved. Hence, it had higher $\overset{.}{\text{V}}$O*˙*_2_ than SPVM where only one leg is involved. In SARV, HAL1, HAL2, KPED, BHUJ, UTP, DHN and PVM it was in the range of 20–26.5% of $\overset{.}{\text{V}}{\text{O}}_{2\max\;}$. In rest of the asanas $\overset{.}{\text{V}}$O*˙*_2_ remained <20% of $\overset{.}{\text{V}}{\text{O}}_{2\max\;}$. This is due to the slow execution of various asanas with adequate rest pause during SAV which is being performed intermittently as a norm in hatha yoga practices.

Now, by studying the dynamics of the various respiratory parameters one can determine the nature of hatha yoga exercises in terms of exercise physiology as compared with conventional physical exercises. During the yogic practice the rise in $\overset{.}{\text{V}}$CO*˙*_2_ was comparatively lower than the corresponding $\overset{.}{\text{V}}$O*˙*_2_ as it happens in conventional dynamic exercises at sub-maximal level. $\overset{.}{\text{V}}$ *˙*E and $\overset{.}{\text{V}}$CO*˙*_2_ increased proportionately with the increase in $\overset{.}{\text{V}}$O*˙*_2_ in all the yoga asanas and in some yoga BMs also. Maximum Respiratory Quotient (RQ) was 0.94 in UTP and it never crossed 1.0 in any of the asana. The lowest range of EQO_2_ as observed among the asanas was from 22 to 24, as in SARV, HAL1, HAL2 and KPED where corresponding $\overset{.}{\text{V}}$O*˙*_2_ was from 0.626 to 0.533 l min^−1^. Closer to that, DHN was having a value of $\overset{.}{\text{V}}$O*˙*_2_ (0.622 l min^−1^) with an EQO_2_ of 26.6. In dynamic exercises with gradually increasing exercise intensity the nadir of EQO_2_ usually remains between 22 and 27 while EQCO_2_ remains between 26 and 30. Thus, a close look into the pattern of changes in EQO_2_ and EQCO_2_ in different asanas reveals that in most of the cases while practicing yogasana, the exercise is well within lactate threshold. From this, there are also indirect indications about normal physiological dead space/tidal volume ratio (VD/VT) and uniform matching of ventilation with respect to perfusion ($\overset{.}{\text{V}}$ *˙*A/$\overset{.}{\text{Q}}$ *˙*) [46]. This non-invasive technique is possibly the only way to establish it as collecting the blood samples during yoga practice would disturb the subject.

In many cases while practicing asanas wherever VT reduced, Fr increased. This helps to maintain the level of $\overset{.}{\text{V}}$ *˙*E as per the requirement of $\overset{.}{\text{V}}$O*˙*_2_ for that particular posture. In PSN, SPVM, PVM, HAL1, HAL2, VAJ, GMK, SARV and KPED values of VT were in the range of 0.57–0.68 l/breath and the corresponding Fr were from 27 to 24 breaths/min. This trend was not common for all the asanas. In this study in YM1, YM2, MYS, SVAJ, GMK2, BHUJ and UTP, VT was in the range of 0.78–0.86 l/breath and corresponding Fr was in the range of 13–22 breaths/min showing increasing trend for both VT and Fr. Brahmachari et al. [47] compared individual yoga postures in yoga proficient subjects and have shown that the increased demand for oxygen in PSN has been met by increasing Fr and increase in VT. The variation in VT in aforementioned asanas is due to restricted movement of the rib cage, respiratory muscles and upper respiratory tract as a result of twisted posture of the neck, chest and upper torso. In a separate study, Rao [28] reported the cost of standing on head was 336 mL $\overset{.}{\text{V}}$O*˙*_2_ or 1.62 kcal min^−1^. In our study values of $\overset{.}{\text{V}}$O*˙*_2_ were 562 mL or 2.73 kcal min^−1^ (Table 2) in SARV which is also a head down posture but where neck is in twisted position. He showed in another study [29] that vital capacity was least in head-down posture (Shirsasana) and it was attributed to strong postural contraction of the respiratory muscles. Moreover, the visceral contents of abdomen slightly shifting towards thoracic cage may also cause certain impediments for diaphragmatic and thoracic respiratory movements. Hemodynamic influence through baroreflex mechanism due to shift of blood to the head and neck region may also influence the VT and Fr due to the interrelated effects of medullary vasomotor areas and respiratory centers. Rai et al. [30–32] studied energy expenditure and ventilatory responses during Siddhasana, Veerasana and MYS. There was moderate rise in $\overset{.}{\text{V}}$ *˙*E and $\overset{.}{\text{V}}$O*˙*_2_ as compared with the SAV. Brahmachari et al. [47] compared individual yogic postures in yoga-proficient subjects and observed that the metabolic cost computed for Siddhasana, PSN and BHUJ were 1.23, 1.45 and 2.62 kcal min^−1^, respectively against the resting SAV value of 1.06 kcal min^−1^. In our study, values of PSN and BHUJ were 2.08 and 2.3 kcal min^−1^, respectively, as against 1.16 kcal min^−1^ in SAV. Highest value of energy cost in this study was 3.02 kcal min^−1^ in HAL2. To our knowledge no study is available to show the energy expenditure separately for each components of yoga practice, that is, for asanas, pranayama and meditation. Average energy expenditure in this program if expressed for its different components, that is, asanas, pranayama and meditation were 2.29, 1.91 and 1.37 kcal min^−1^, respectively. Same was reported by other investigators [48] during the practice of yoga as 3.7 kcal min^−1^, but the values of specific yoga exercises were not available. Clay et al. [35] and Hagins et al. [36] showed that the mean caloric expenditure in the entire yoga session was 2.23 and 3.2 kcal min^−1^ respectively. Those studies particularly of Hagins et al. [36] included mostly Sun salutation (Suryanamaskar, a combination of yogic physical postures practiced in a sequence continuously), which covered 50–60% of entire yoga session. We have already conducted a separate study on Suryanamaskar [34]. This part of the study has focused on other aspects of yogic practices, that is, asanas, different BMs and meditation excluding the Suryanamaskar because it is generally not prescribed for the patients or people with poor body flexibility or older people but is suitable for comparatively physically fit individuals.

In our earlier study, Sinha et al. [34] reported energy expenditure of 3.79 kcal min^−1^ in Suryanamaskar. Hagins et al. [36] also showed the energy expenditure in the entire yoga session (comprising Suryanamaskar) to be 3.2 kcal min^−1^, which is very much similar to the values found by us. The mask used for the measurement of $\overset{.}{\text{V}}$O*˙*_2_ in this study was very comfortable to the subjects. Hence, the issue raised by Hagins et al. [36] that the differences in energy expenditure between their study and the study of Clay et al. [35] (with mask, showing lower energy expenditure) appears to be insignificant in this regard.

Energy expenditure during cycling (5.5 mph) is 3.8 kcal min^−1^ and walking on treadmill (2.0 mph) is 3.1 kcal min^−1^ [48]. While comparing these with yoga the average energy expenditure during the practice of asanas in our study shows 60.3 and 73.8% of that of cycling and walking, respectively. Brahmachari et al. [49] also studied similarly on three hatha yoga postures, that is, Padmasana, Kurmasana and Ustrasana. Properly performed asana, that is, the posture involving more of cerebellar activity exhibits relaxed condition of the related muscles which otherwise show increased electrical activity if the same condition is maintained with the help of isometric contraction (exercise involving more of motor cortex) [33]. Hence, energy cost for performing an asana depends on two factors. First, it is the involvement of total muscle mass and secondly on the conditioning that is, how much optimum utilization of the muscles is being done in these practices. Intensity of exercise in terms of MET during conventional physical exercise as in cycling (<10 mph) and walking (3.5 mph) was reported to be 4.0 MET [26]. The highest value in our study was 2.19 MET in DHN and HAL-2 which are 54.7 and 62.6% of cycling and walking respectively.

Like % $\overset{.}{\text{V}}{\text{O}}_{2\max\;}$ the intensity of exercise when expressed in MET shows in this study that hatha yoga practice comes under the category of moderate physical exercise and its range never goes beyond 2.19 MET. To perform higher intensity of exercise there is only one possibility, which is to increase the frequency and duration of specific yogic practice that have higher level of MET.

In the same subjects during the entire yoga session the maximal heart rate (MHR) was in the range of 97.9 ± 12.7 to 102.1 ± 18.9 beats/min and their mean MHR in bicycle exercise was 187.2 ± 9.3 beats/min. So, in the present study subjects could attain only 52.3–54.5% of their MHR (our unpublished observation). This is even lower than the similar kind of values reported by Clay et al. [35]. They have shown that during 30 min hatha yoga session in women (age 19–40 years), heart rate elevated up to 56.9% of MHR. According to the American College of Sports Medicine guideline the minimum average heart rate value to be achieved in an exercise session for fitness gain should be 55% of MHR [38] for sedentary and unfit individuals. In our study these values are slightly lower than the %MHR as reported by Hagins et al. [36] and Clay et al. [35]. The difference between those studies and the present study is that there Suryanamaskar was a major component of yoga session; while in the present study Suryanamaskar was not at all incorporated. The criteria of achieving particular level of %MHR may not be always reliable as the yogic postural exercises (asanas) also have isometric nature, as Caroll et al. [37] could not find any correlation between the $\overset{.}{\text{V}}$O*˙*_2_ and heart rate in yogic exercises.

In spite of the lower level of exercise intensity, yoga practices have the potentiality to maintain the physical performance status of an individual. Our previous study [8] in this regard showed that a group of soldiers practicing Hatha yoga instead of conventional physical exercise could improve $\overset{.}{\text{V}}{\text{O}}_{2\max\;}$. Nayar et al. [50] showed that physical performance at sub-maximal level improves after yoga practices. The works of Raju et al. [46, 47] also indicated the physical performance improvement in the similar way. Pollen et al. [16] showed that even in heart failure patients peak $\overset{.}{\text{V}}$O*˙*_2_ and total exercise time increased after yoga training. Possibly, different combinations of yoga practices along with other dynamic exercises have the potentialities to increase the physical performance in different degrees. Hatha yoga BMs are having the potentiality to train the respiratory system in such a way that it helps an individual to cope with the respiratory demand with respect to higher intensity of exercise also. In yoga BMs respiratory rate increased as high as it happens in maximal aerobic exercise as it was observed in KB in our study. But these maneuvers with voluntary deep breathing (BHAR, KAKI and YONI) help to achieve a state wherein an individual's respiratory rate becomes relatively lower with respect to their increasing VT by allowing greater time for diffusion of oxygen in the respiratory system. The studies of Bernardi et al. [3, 44] and Stanescu et al. [2] have indicated the same. We [45] have seen that yogic BMs possibly help to improve cardiac stroke volume which also may be an important factor to improve physical performance. In our previous studies it has also been observed that the yogic practices improves the muscular efficiency in terms of reduced electromyography amplitude and endurance time in static isometric exercise [7] and also body flexibility [9] apart from its famous psycho-physiological benefits [1, 21–24].

**Figure 3:**
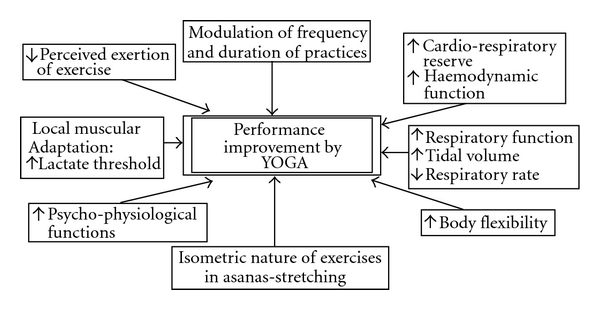
Possible factors responsible for physical performance improvement by yoga though it is low to moderate intensity of exercise in terms of % $\overset{.}{\text{V}}{\text{O}}_{2\max\;}$, energy cost, MET and Percent MHR as observed in this study. Integratd from the findings of this study and those of [2, 7–10, 16, 34, 40, 43–45].

There is a possibility in hatha yoga to improve physical performance, in spite of the exercise stimulus being of lower magnitude as compared with conventional dynamic exercises (Figure 3). The underlying mechanisms including the cardiovascular and peripheral metabolic ones need to be explored systematically further. The study by Wallace et al. [5] has shown that yoga-proficient subjects could achieve the hypo-metabolic state in their subjects during meditation. In the present study oxygen consumption also reduced during meditation indicating better relaxed state. In this study, BMs with a longer breath hold time such as Kumbhaka were not administered. This was done in spite of the subjects of this study being “yoga instructors" as we wanted to compare the values with common subjects who may not be yoga proficient and might not practice “Kumbhaka". It requires lot of caution and longer time to learn to perform it perfectly. The results of the study may be used for the common people.

In different yoga programs with various combinations of different components of yogic practices, with regulated duration and frequency of specific yogic practices the physical performance improvement can be achieved. Different combinations of yogic practices may be incorporated in physical fitness program based on the situation both in normal and diseased individuals. All practices should not be performed by everybody. The comparatively physically fit individuals may practice all the practices by increasing the frequency and duration of individual components. On the contrary, for sedentary and diseased individuals asanas with the lower or moderate intensity of exercise without any complicated postures (asanas) should be preferred. One has to be selective to choose specific yoga practices in case of sedentary and diseased persons to accrue the benefits of very wide range of physical [7–9] and psycho-physiological ones [1, 10] including the preventive, prophylactic and curative aspects for various diseases [11–24] by practicing it with low level of energy cost/exercise intensity (less stress). The very convenience of hath yoga practice is this.

So, this study provides basic data on $\overset{.}{\text{V}}$O*˙*_2_, energy cost, exercise intensity in terms of $\overset{.}{\text{V}}{\text{O}}_{2\max\;}$, and MET along with the dynamics of relevant respiratory parameters for individual components of a yoga session and also for the entire session as a whole. In general, the values of energy expenditure and intensity of exercise indicate that in yoga, exercise stimulus may not be that high to improve physical performance. Still, our previous studies and also of others have indicated the improvement of physical and mental performance by its practice. Therefore, hatha yoga practice may be a more energy-efficient way to accrue the meaningful physiological benefits for the general public as well as for people who are not able to undergo high intensity exercises as in diseased individuals. It may be performed by selecting appropriate yoga postures/practices with due consideration on intensity of exercise and duration of practice based on the physical fitness of the individual.

## Funding

Grant number 9-16/95-96/CCRYN/Yoga/915 of Central Council of Research in Yoga and Naturopathy of Ministry of Health and Family Welfare, Govt of India.

## Figures and Tables

**Table 1:** Particulars about *yoga* practices.

No.	Asanas	Time (in minutes)	Remarks
1	KB	2	—
2	YM 1 and YM 2	2	two postures, each two times
3	PSN	2	—
4	SPVM	1	—
5	PVM	1	—
6	DHN	2	1/2 min rest after first min
7	MYS	2	1/2 min rest after first min
8	VAJ	1	—
9	SVAJ	1	—
10	GMK1, GMK2		2
11	SARV		1
12	HAL1	1	—
13	HAL2	1	—
14	KPED		1
15	BHUJ	2	1/2 min rest after first min
16	UTP	2	1/2 min rest after first min
17	SAV^a^	after every asanas	
18	BHAS	2	1/2 min rest
19	KAKI	1	5–7 s rest after each 1/2 min practice
20	YONI	1	5–7 s rest after each 1/2 min practice
21	BHRA	2	—
22	OM MED	3	—
23	MED	2	
24	SUKH^a^	after pranayama, kaki mudra, yoni mudra	—

^a^Practiced until steady resting values in heart rate and $\overset{.}{\text{V}}$O*˙*_2_ were achieved.

**Table 2:** Metabolic rate, energy cost, % $\overset{.}{\text{V}}{\text{O}}_{2\max\;}$ and various respiratory parameters during the actual practice of asanas.

	Parameters										
	$\overset{.}{\text{V}}$O_2_ (l min^−1^)	$\overset{.}{\text{V}}$ *˙*CO_2_ (l min^−1^)	$\overset{.}{\text{V}}$ *˙*E (l min^−1^)	Fr (breaths/min)	VT (l/breath)	EQO_2_	EQCO_2_	RQ	MET	Energy cost (kcal)	% $\overset{.}{\text{V}}{\text{O}}_{2\max\;}$
YM1	0.401 ± *0.12*	0.331 ± 0.10	10.4 ± 3.16	14.0 ± 5.23	0.83 ± 0.36	22.8 ± 4.71	27.7 ± 2.95	0.82 ± 0.11	1.37 ± 0.41	1.93 ± 0.60	14.1 ± 2.4
YM2	0.369 ± *0.07*	0.302 ± 0.05	9.3 ± 1.90	13.0 ± 5.92	0.86 ± 0.58	21.2 ± 3.53	26.5* ± 2.54	0.80 ± 0.09	1.30 ± 0.32	1.80 ± 0.33	17.4 ± 2.34
PSN	0.433 ± *0.13*	0.371 ± 0.11	11.8 ± 3.24	20.0 ± 5.01	0.61 ± 0.13	23.9 ± 4.72	27.6 ± 5.72	0.86 ± 0.08	1.49 ± 0.43	2.08 ± 0.61	15.7 ± 3.8
SPVM	0.391 ± *0.09* ^†^	0.352 ± 0.07	11.6 ± 2.47	20.0 ± 5.35	0.67 ± 0.30	26.2 ± 5.27	29.1 ± 4.27	0.90 ± 0.13	1.34 ± 0.28	1.87 ± 0.43	15.9 ± 2.93
PVM	0.542* ± *0.12* ^†^	0.451 ± 0.11	14.9 ± 3.71	24.0* ± 6.18	0.68 ± 0.26	24.2 ± 4.29	29.0 ± 3.95	0.83 ± 0.12	1.88 ± 0.49	2.62 ± 0.57	21.0 ± 5.51
DHN	0.624* ± *0.07*	0.543 ± 0.09	17.9* ± 2.32	24.0* ± 4.33	0.73 ± 0.15	26.6 ± 2.76	28.4 ± 5.83	0.89 ± 0.13	2.19* ± 0.49	3.03* ± 0.35	26.5 ± 6.02
MYS	0.471 ± *0.12*	0.412 ± 0.09	13.7 ± 2.90	17.0 ± 4.70	0.79 ± 0.19	26.6 ± 5.03	28.9 ± 3.75	0.89 ± 0.13	1.63 ± 0.44	2.28 ± 0.59	19.8 ± 4.89
VAJ	0.372 ± *0.09* ^‡^	0.321 ± 0.07	10.9 ± 2.23	17.0 ± 3.76	0.65 ± 0.15	26.3 ± 4.25	29.8 ± 3.51	0.88 ± 0.07	1.28 ± 0.26	1.81 ± 0.43	15.5 ± 2.78
SVAJ	0.433 ± *0.11* ^‡^	0.392 ± 0.11	13.3 ± 3.39	21.0 ± 4.22	0.72 ± 0.22	26.8 ± 4.62	29.8 ± 3.64	0.91 ± 0.09	1.49 ± 0.31	2.09 ± 0.54	17.5 ± 2.35
GMK1	0.421 ± *0.10*	0.362 ± 0.09	12.7 ± 2.79	20.0 ± 4.53	0.65 ± 0.16	26.8 ± 4.38	30.5 ± 3.81	0.88 ± 0.10	1.45 ± 0.39	2.02 ± 0.50	18.4 ± 3.68
GMK 2	0.521 ± *0.10*	0.441 ± 0.09	14.9 ± 3.11	22.0 ± 5.2	0.70 ± 0.16	25.5 ± 3.54	29.9 ± 3.66	0.85 ± 0.06	1.82 ± 0.44	2.45 ± 0.49	22.8 ± 5.38
SARV	0.562* ± *0.14*	0.452 ± 0.09	15.1 ± 2.40	25.0* ± 5.23	0.61 ± 0.10	22.3 ± 3.48	27.4 ± 3.35	0.82 ± 0.09	1.94 ± 0.46	2.73 ± 0.69	22.7 ± 5.5
HAL 1	0.571* ± *0.18*	0.513 ± 0.14	16.9* ± 3.79	27.0* ± 4.23	0.61 ± 0.13	24.3 ± 2.57	28.8 ± 5.32	0.82 ± 0.08	2.03* ± 0.68	2.79 ± 0.89	25.9 ± 7.68
HAL 2	0.631* ± *0.19*	0.524 ± 0.13	16.7* ± 3.24	27.0* ± 6.21	0.63 ± 0.15	22.0 ± 3.78	27.1 ± 4.33	0.81 ± 0.09	2.19* ± 0.68	3.05* ± 0.91	24.6 ± 7.77
KPED	0.571* ± *0.17*	0.471 ± 0.10	15.6 ± 3.20	27.0* ± 6.29	0.57* ± 0.11	22.3 ± 3.27	27.43 ± 4.16	0.79 ± 0.08	2.00* ± 0.62	2.79 ± 0.81	22.7 ± 6.05
BHUJ	0.471 ± *0.08*	0.412 ± 0.08	13.8 ± 2.70	19.0 ± 5.47	0.73 ± 0.19	26.6 ± 4.47	30.2 ± 4.27	0.87 ± 0.09	1.66 ± 0.37	2.30 ± 0.36	20.7 ± 4.51
UTP	0.502 ± *0.10*	0.451 ± 0.13	16.1* ± 3.34	21.0 ± 4.75	0.71 ± 0.17	27.8* ± 5.26	29.5 ± 3.90	0.98 ± 0.10	1.76 ± 0.48	2.42 ± 0.50	23.9 ± 5.04
SAV	0.243 ± *0.04*	0.222 ± 0.04	6.9 ± 1.43	13.0 ± 3.24	0.54 ± 0.15	24.7 ± 4.61	27.7 ± 4.07	0.89 ± 0.10		1.16	9.9 ± 1.21

Data are mean ± SD.

*indicates asanas (posture) statistically significant (*P* <  .05 and above) as compared with others.

^†^ significantly (*P* <  .001) different.

^‡^significantly (*P* <  .05) different among themselves.

**Table 3:** Metabolic rate, energy cost and various respiratory parameters during the actual practice of BMs as compared with those of SUKH.

Parameters	KB	BHAS	KAKI	YONI	BHRA	SUKH
$\overset{.}{\text{V}}$O*˙*_2_ (l min^−1^)	0.451* ± *0.15* ^†^	0.262 ± *0.04*	0.514* ± *0.16* ^†^	0.503* ± *0.18* ^†^	0.331 ± *0.07*	0.312 ± *0.07*
$\overset{.}{\text{V}}$CO_2_ (l min^−1^)	0.714 ± 0.34	0.174 ± 0.04	0.424 ± 0.12	0.381 ± 0.15	0.281 ± 0.06	0.242 ± 0.07
$\overset{.}{\text{V}}$ *˙*E (l min^−1^)	53.5* ± 15.47	6.6 ± 1.38	12.7 ± 4.02	11.7 ± 3.79	8.9 ± 2.29	8.8 ± 2.58
Fr (breaths/min)	65.0* ± 27.41	12.0 ± 4.66	14.0 ± 5.33	13.0 ± 4.90	10.0 ± 6.35	16.0 ± 4.86
VT (l/breath)	0.64 ± 0.31	0.59 ± 0.21	0.97 ± 0.59	0.77 ± 0.37	1.41* ± 1.27	0.59 ± 0.15
EQO_2_	79.0* ± 33.62	21.5 ± 5.49	22.1 ± 5.17	23.2 ± 5.84	24.5 ± 5.39	24.7 ± 5.71
EQCO_2_	60.8* ± 13.90	27.9 ± 11.99	27.9 ± 5.31	28.7 ± 5.84	28.6 ± 3.11	31.1 ± 5.91
RQ	1.60 ± 0.52	0.64 ± 0.14	0.79 ± 0.10	0.81 ± 0.10	0.85 ± 0.11	0.78 ± **0.1**
MET	1.55* ± 0.55	0.92 ± 0.21	1.78* ± 0.61	1.72* ± 0.56	1.17 ± 0.34	1.07 ± 0.28
Energy cost (kcal)	2.17* ± 0.73	1.28 ± 0.21	2.50* ± 0.80	2.45* ± 0.90	1.61 ± 0.34	1.49 ± 0.34

Data are mean ± SD.

*indicates BMs statistically significant (*P* < .05 and above) as compared with others.

^†^ significantly different compared to SUKH.

**Table 4:** Metabolic rate, energy cost and various respiratory parameters during the actual practice of meditation as compared with those of SAV and SUKH.

Parameters	OM MED	MED	SAV	SUKH
$\overset{.}{\text{V}}$O_2_ (l min^−1^)	0.301* ± *0.08*	0.272 ± *0.05*	0.243* ± *0.04*	0.312 ± *0.07* ^†^
$\overset{.}{\text{V}}$CO_2_ (l min^−1^)	0.272* ± 0.11	0.213* ± 0.06	0.222* ± 0.04	0.242 ± 0.07
$\overset{.}{\text{V}}$ *˙*E (l min^−1^)	8.5* ± 2.42	6.9* ± 1.62	6.9* ± 1.43	8.8* ± 2.58^†^
Fr (breath/min)	10.0* ± 5.82	13.0 ± 4.36	13.0 ± 3.24	16.0* ± 4.86
VT (l/breath)	1.28* ± 1.14	0.61* ± 0.26	0.54* ± 0.15	0.59* ± 0.15
EQO_2_	25.2* ± 6.43	22.3* ± 4.39^†^	24.7 ± 4.61	24.7* ± 5.71
EQCO_2_	29.1* ± 3.51^†^	29.9 ± 4.1^†^	27.7 ± 4.07	31.1* ± 5.91^†^
RQ	0.85* ± 0.16	0.74* ± 0.09^†^	0.89 ± 0.1	0.78* ± 0.1^†^
MET	1.04* ± 0.29	0.93 ± 0.23	0.83* ± 0.12	1.07 ± 0.28^†^
Energy cost (kcal)	1.46* ± 0.38	1.29* ± 0.27^†^	1.16* ± 0.19	1.49 ± 0.34^†^

Data are mean ± SD.

*indicates statistically significant difference (*P* <  .05 and above) as compared to the corresponding asterisk-marked ones except in VCO_2_: MED Versus SAV; VE: MED Versus SAV, OMMED Versus SUKH; VT: MED Versus SUKH, SAV Versus MED, SAV Versus SUKH; EQO_2_: OMMED Versus SUKH; RQ: OMMED Versus SUKH.

^†^significantly (*P* <  .01) than SAV.

## References

[1] Raub JA (2002). Psychophysiologic effects of *hatha Yoga* on musculoskeletal and cardiopulmonary function: a literature review. *Journal of Alternative and Complementary Medicine*.

[2] Stanescu DC, Nemery B, Veriter C, Marechal C (1981). Pattern of breathing and ventilatory response to CO2 in subjects practicing *hatha yoga*. *Journal of Applied Physiology Respiratory Environmental and Exercise Physiology*.

[3] Bernardi L, Sleight P, Bandinelli G (2001). Effect of rosary prayer and *yoga* mantras on autonomic cardiovascular rhythms: comparative study. *BMJ*.

[4] Chaya MS, Nagendra HR (2008). Long—term effect of yogic practices on diurnal metabolic rates of healthy subjects. *International Journal of Yoga*.

[5] Wallace RK, Benson H (1972). The physiology of meditation. *Scientific American*.

[6] Danucalov MA, Simóes RS, Kozasa EH, Leite JR (2008). Cardio-respiratory and metabolic changes during yoga sessions: the effects of respiratory exercises and meditation practices. *Applied Psychophysiology and Biofeedback*.

[7] Ray US, Hegde KS, Selvamurthy W (1986). Improvement in muscular efficiency as related to a standard task after yogic exercises in middle aged men. *Indian Journal of Medical Research*.

[8] Ray US, Sinha B, Tomer OS, Pathak A, Dasgupta T, Selvamurthy W (2001). Aerobic capacity & perceived exertion after practice of *hatha yogic* exercises. *Indian Journal of Medical Research*.

[9] Ray US, Hegde KS, Selvamurthy W (1983). Effects of yogic *asanas* and physical exercises on body Flexibility in middle aged men. *Yoga Review*.

[10] Ray US, Mukhopadhyaya S, Purkayastha SS (2001). Effect of yogic exercises on physical and mental health of young fellowship course trainees. *Indian Journal of Physiology and Pharmacology*.

[11] Yang K (2007). A review of yoga programs for four leading risk factors of chronic diseases. *Evidence-Based Complementary and Alternative Medicine*.

[12] Bijlani RL, Vempati RP, Yadav RK (2005). A brief but comprehensive lifestyle education program based on *yoga* reduces risk factors for cardiovascular disease and diabetes mellitus. *Journal of Alternative and Complementary Medicine*.

[13] Cohen DL, Bloedon LT, Rothman RL *Iyenger yoga* versus enhanced usual care on blood pressure in patients with prehypertension to stage 1 hypertension a randomized controlled trial.

[14] McCaffrey R, Ruknui P, Hatthakit U, Kasetsomboon P (2005). The effects of *yoga* on hypertensive persons in Thailand. *Holistic nursing practice*.

[15] Ornish D, Brown SE, Scherwitz LW (1990). Can lifestyle changes reverse coronary heart disease?. *Lancet*.

[16] Pullen PR, Nagamia SH, Mehta PK (2008). Effects of *Yoga* on Inflammation and Exercise Capacity in Patients With Chronic Heart Failure. *Journal of Cardiac Failure*.

[17] Agte VV, Tarwadi K (2004). *Sudarshan kriya yoga* for treating type 2 diabetes: a preliminary study. *Alternative and Complementary Therapies*.

[18] Gordon LA, Morrison EY, McGrowder DA (2008). Effect of exercise therapy on lipid profile and oxidative stress indicators in patients with type 2 diabetes. *BMC Complementary and Alternative Medicine*.

[19] Nagarathna R, Nagendra HR (1985). *Yoga* for bronchial asthma: a controlled study. *British Medical Journal*.

[20] Skoro-Kondza L, Tai SS, Gadelrab R, Drincevic D, Greenhalgh T (2009). Community based *yoga* classes for type 2 diabetes: an exploratory randomised controlled trial. *BMC Health Services Research*.

[21] Brown RP, Gerbarg PL (2005). *Sudarshan Kriya* yogic breathing in the treatment of stress, anxiety, and depression: part I—neurophysiologic model. *Journal of Alternative and Complementary Medicine*.

[22] Butler LD, Waelde LC, Hastings TA, Chen XH, Symons B, Marshall J (2008). Meditation with *yoga*, group therapy with hypnosis and psycho education for long-term depressed mood: a randomized pilot trial. *Journal of Clinical Psychology*.

[23] Shapiro D, Cook IA, Davydor DM, Ottaviani C, Leuohter A, Abrams M (2007). *Yoga* as a complementary treatment of Depression: effects of traits and moods on treatment outcome. *Evidence-Based Complementary and Alternative Medicine/source*.

[24] Khalsa SBS (2004). Treatment of chronic insomnia with yoga: a preliminary study with sleep-wake diaries. *Applied Psychophysiology Biofeedback*.

[25] Tindle HA, Davis RB, Phillips RS, Eisenberg DM (2005). Trends in use of complementary and alternative medicine by us adults: 1997–2002. *Alternative Therapies in Health and Medicine*.

[26] Ainsworth BE, Haskell WL, Whitt MC (2000). Compendium of physical activities: an update of activity codes and MET intensities. *Medicine and Science in Sports and Exercise*.

[27] Salgar DC, Bishen VS, Jinturkar MJ (1975). Effect of *Padmasana*—a yogic exercise on muscular efficiency. *Indian Journal of Medical Research*.

[28] Rao S (1962). Metabolic conditions of head stand posture. *Journal of Applied Physiology*.

[29] Rao S (1968). Respiratory responses to headstand posture. *Journal of applied physiology*.

[30] Rai L, Ram K (1992). Energy expenditure and ventilatory responses during *Matsyasana*—a yogic backward bending posture. *Yoga Mimansa*.

[31] Rai L, Ram K (1993). Energy expenditure and ventilatory responses during *Veerasana*— a yogic standing posture. *Indian Journal of Physiology & Pharmacology*.

[32] Rai L, Ram K, Kant U, Madan SK, Sharma SK (1994). Energy expenditure and ventilatory responses during *Sidhasana*—a yogic seated posture. *Indian Journal of Physiology & Pharmacology*.

[33] Karambelkar PV, Bhole MV, Gharote ML (1969). Muscle activity in some *asanas*. *Yoga Mimansa*.

[34] Sinha B, Ray US, Pathak A, Selvamurthy W (2004). Energy cost and cardio respiratory changes during the practices of *Suryanamaskar*. *Indian Journal of Physiology & Pharmacology*.

[35] Clay CC, Lloyd LK, Walker JL, Sharp KR, Pankey RB (2005). The metabolic cost of *hatha yoga*. *Journal of Strength and Conditioning Research*.

[36] Hagins M, Moore W, Rundle A (2007). Does practicing *hatha yoga* satisfy recommendations for intensity of physical activity which improves and maintains health and cardiovascular fitness?. *BMC Complementary and Alternative Medicine*.

[37] Carroll J, Blansit A, Otto RM, Wygand JW (2003). The metabolic requirements of *Vinyasa Yoga*. *Medicine & Science in Sports & Exercise*.

[38] DiCarlo LJ, Sparling PB, Hinson BT, Snow TK, Rosskopf LB (1995). Cardiovascular, metabolic and perceptual responses to *hatha Yoga* standing poses. *Medicine, Exercise, Nutrition and Health*.

[39] Saraswati SS (1997). *Asana, Pranayama, Mudra, Bandha*.

[40] Sivananda SS (1981). *Hatha Yoga. Science of Yoga*.

[41] Sloan AW (1967). Estimation of body fat in young men. *Journal of Applied Physiology*.

[42] De vries HA (1986). *Excercise Metabolism. Physiology of Exercise*.

[43] Raju PS, Madhavi S, Prasad KVV (1994). Comparison of effects of *yoga* and physical exercise in athletes. *Indian Journal of Medical Research*.

[44] Bernardi L, Spadacini G, Bellwon J, Hajric R, Roskamm H, Frey AW (1998). Effect of breathing rate on oxygen saturation and exercise performance in chronic heart failure. *Lancet*.

[45] Ray US, Tomer OS Cardiovascular haemodynamic changes during the practice of yogic breathing maneuvers.

[46] Wasserman K, Whipp BJ, Sue DY, Casaburi RC, Hansen JE, Harris M (1994). Measurement during integrative cardiopulmonary exercise testing. *Principal of Exercise Testing and Interpretation Exercise*.

[47] Brahmachari D, Vachani V, Sahani S, Ram K, Rai L (1989). A comparative study of some individual yogic postures on ventilatory responses in *yoga* proficient subjects. *Journal of Research and Education in Indian Medicine*.

[48] Mc Ardle WD, Katch VL (1996). Appendix-D, Energy expenditure invarious activities. *Exercise Physiology*.

[49] Brahmachari D, Vachani V, Sahani S, Ram K, Rai L (1989). Study of the effect of *Padmasana, Kurmasana* and *Ushtrasana* on cardio-ventilatory functions in normal persons. *Journal of Research and Education in Indian Medicine*.

[50] Nayar HS, Mathur RM, Sampath Kumar R (1975). Effects of yogic exercises on human physical efficiency. *Indian Journal of Medical Research*.

